# A dataset for fault detection and diagnosis of an air handling unit from a real industrial facility

**DOI:** 10.1016/j.dib.2023.109208

**Published:** 2023-05-09

**Authors:** Michael Ahern, Dominic T.J. O'Sullivan, Ken Bruton

**Affiliations:** a Intelligent Efficiency Research Group (IERG), Department of Civil and Environmental Engineering, University College Cork, T12 CY82 Cork, Ireland; b MaREI Centre, Environmental Research Institute, University College Cork, T12 CY82 Cork, Ireland

**Keywords:** Real data, HVAC data, Detection, Time series

## Abstract

This dataset was collected for the purpose of applying fault detection and diagnosis (FDD) techniques to real data from an industrial facility. The data for an air handling unit (AHU) is extracted from a building management system (BMS) and aligned with the Project Haystack naming convention. This dataset differs from other publicly available datasets in three main ways. Firstly, the dataset does not contain fault detection ground truth. The lack of labelled datasets in the industrial setting is a significant limitation to the application of FDD techniques found in the literature. Secondly, unlike other publicly available datasets that typically record values every 1 min or 5 min, this dataset captures measurements at a lower frequency of every 15 min, which is due to data storage constraints. Thirdly, the dataset contains a myriad of data issues. For example, there are missing features, missing time intervals, and inaccurate data. Therefore, we hope this dataset will encourage the development of robust FDD techniques that are more suitable for real world applications.

**Specifications Table**

**Table:** 

Subject	Mechanical Engineering
Specific subject area	Industrial air handling unit fault detection and diagnosis
Type of data	Table
How the data were acquired	Extraction of data from the industrial facilities Building Management System (BMS) and cloud-based storage system.
Data format	Raw
Description of data collection	The data available for an air handling unit was obtained from the BMS and the cloud based storage system. The data was then anonymized and pre-processed to align with standardized naming conventions.
Data source location	Country: Ireland
Data accessibility	Repository name: Mendeley Data Data identification number: 10.17632/8 × 62ntvrg7.2 Direct URL to data: https://data.mendeley.com/datasets/8 × 62ntvrg7/2
Related research article	M. Ahern, D. T. J. O. Sullivan, and K. Bruton, “Implementation of the IDAIC framework on an air handling unit to transition to proactive maintenance,” *Energy Build.*, vol. 284, p. 112,872, 2023.

## Value of the Data

• This dataset addresses the lack of AHU data from real industrial facilities. This data contains many instances of real world data issues such as Broken data (e.g. missing data points), Bad Quality data (e.g. noisy sensor data) and Background issues (e.g. lack of data labeling).
• Both industrial practitioners and those from the academic community may benefit from this dataset.
• As the dataset is unlabeled, an integrated knowledge-based and data-driven approach is recommended to investigate the importance of trends and patterns in the data in relation to detecting and diagnosing faults.

## 1 Objective

This dataset has been generated to expose the wider academic community to the issues encountered when working with real world datasets. There are a variety of issues with this dataset that have been documented in the companion research article [1], such as erroneous timestamps, spike faults, missing data, etc. One of the main differentiating points with this dataset is the lack of ground truth information, such as the unknown presence or absence of faults, lack of fault intensity and lack of fault duration information. This will hopefully encourage other researchers to develop robust techniques that may distinguish impactful faults from noise in the data using domain knowledge or otherwise. Furthermore, it is envisaged that future FDD efforts will require a highly interpretable justification of the faults it identifies. This is a necessary direction for future research to translate the progress made on simulated and experimental datasets to the real world.

## 2 Data Description

The data [2] contains time series readings, such as control signals and temperature sensor readings, at various locations throughout the AHU as in Figure 2 in the research article [1]. The data may be used to detect faults in AHUs such as sensor faults, controller faults, equipment faults and actuator faults.

The data is stored as a series of csv files on the BMS, referred to as log files, which contain the data relating to one sensor or control signal. In consultation with the onsite facilities team, the metadata for each log file is analyzed to determine the sensor location in the AHU. This information is then standardized to align to the Project Haystack [3] naming convention. For example, the log file relating to the return air temperature sensor is named using a bespoke combination of letters and numbers on the BMS. This information is then contextualized in accordance with the State of Utah Haystack Tagging Reference Model Example [4], becoming “RaTemp”. The remaining data points presented in Table 1 follow a similar process.

**Table 1:** Description of data points.

Tag Value	Description	Unit	Sampling Rate
RaTemp	Return air temperature	°C	900s
RaFanPower	Return air fan power	kWh	900s
EaDmprPos*	Exhaust air damper control signal	%	900s
RaDmprPos*	Return air damper control signal	%	900s
OaTemp	Outside air temperature sensor	°C	900s
OaDmprPos	Outside air damper control signal	%	900s
MaTemp	Mixed air temperature	°C	900s
HWVlvPos	Hot water valve control signal	%	900s
HCALTemp	Air leaving hot water coil temperature	°C	900s
ChWVlvPos	Chilled water valve control signal	%	900s
CCALTemp	Air leaving chilled water coil temperature	°C	900s
DaFanPower	Supply air fan power	kWh	900s
DaTemp	Supply air temperature	°C	900s
OaTemp_WS	Outside air temperature of nearby weather station	°C	900s
ReHeatVlvPos_1	Reheat hot water valve control signal for zone 1	%	900s
ReHeatVlvPos_2	Reheat hot water valve control signal for zone 2	%	900s
ZoneDaTemp_1	Zone supply air temperature for zone 1	°C	900s
ZoneDaTemp_2	Zone supply air temperature for zone 2	°C	900s
ZoneTemp_1	Average zone 1 air temperature	°C	900s
ZoneTemp_2	Average zone 2 air temperature	°C	900s

As discussed in the research article [1], there is missing data in the log files. This missing data has been backfilled with data from a cloud based data storage system that was connected in 2020. The two datasets were merged using the condition that if the log file data is missing, it would be updated using the values from the cloud based system. The data from this system also included data points that were not available on the BMS such as data from a nearby weather station (OaTemp_WS) and fan power data (RaFanPower & DaFanPower).

## 3 Experimental Design, Materials and Methods

The purpose of this dataset is to expose the academic community to the real world challenges of FDD in the industrial setting. As the industry is regulated, there is no opportunity to manually impose faults and analyze the systems response. Therefore, it is not only difficult to determine the occurrence of a fault, but also determine the different fault types or fault intensities. The approach in the accompanying research article [1] to address this challenge utilizes an integration-based adaptation of the CRISP-DM process model [5] to leverage domain knowledge and data analytics to analyze the data. There is further scope for other researchers to use data-driven techniques to identify abnormal behavior in this dataset and provide a justification of the diagnosis to improve the interpretability of the results.

While there is a lack of documentation of maintenance activities carried out during the time period of the dataset, regular meetings with the facilities team led to two main activities taking place. Firstly, on the 02/12/2021, a review of the control strategy of the AHU took place in which the outside air damper control was returned to normal operation after a period of being fixed at 100% open as a safety precaution during the COVID-19 pandemic. Secondly, on the 01/05/2022, a shutdown occurred at the industrial facility whereby the heating coil control valve and cooling coil control valve were replaced.

## Ethics Statements

Not applicable.

## CRediT authorship contribution statement

**Michael Ahern:** Data curation, Writing – original draft, Investigation, Conceptualization, Methodology, Writing – review & editing. **Dominic T.J. O'Sullivan:** Writing – review & editing, Supervision. **Ken Bruton:** Conceptualization, Methodology, Writing – review & editing, Supervision.

## Declaration of Competing Interest

The authors declare that they have no known competing financial interests or personal relationships that could have appeared to influence the work reported in this paper.

## Data Availability

A dataset for fault detection and diagnosis of an air handling unit from a real industrial facility (Original data) (Mendeley Data).
